# Minimum important difference of the ICIQ-UI SF score after self-management of urinary incontinence

**DOI:** 10.1186/s12905-024-02947-x

**Published:** 2024-02-14

**Authors:** Emma Nyström, Ina Asklund, Anna Lindam, Eva Samuelsson

**Affiliations:** 1 Department of Public Health and Clinical Medicine, Umeå University, Umeå, Sweden; 2 Department of Public Health and Clinical Medicine, Unit of Research, Education, and Development - Östersund, Umeå University, Umeå, Sweden

**Keywords:** Urinary incontinence, PGI-I; minimum important difference, ICIQ-UISF, Pelvic floor muscle training, Mobile app

## Abstract

**Background:**

This study aimed to evaluate clinically relevant improvement after conservative self-management of urinary incontinence via a mobile app. It further aimed to establish Minimum Important Differences (MIDs) based on the severity and type of urinary incontinence.

**Methods:**

Data was collected in a prospective cohort study that evaluated the freely available app Tät®. The app provided pelvic floor muscle training (PFMT) and life-style advice. Non-pregnant, non-postpartum women (≥ 18 years) who downloaded the app to treat urinary incontinence were included, if they completed the Patient Global Impression of Improvement (PGI-I) question at the 3-month follow-up (*n* = 1,733). Participants answered the International Consultation on Incontinence Questionnaire (ICIQ-UI SF) at baseline and after 3 months. The score change was analysed for correlation (Spearman) with the PGI-I. We then analysed one-way ANOVAs to determine whether there were significant differences between the groups based on the answers to the PGI-I. The MID was set to the mean change of the group that selected the answer “a little better” to the PGI-I question.

**Results:**

The one-way ANOVA showed significant differences between PGI-I groups (*p* < 0.001). The MID for the general group was set to 1.46 (95% Confidence Interval [CI] 1.26–1.67). In the sub-group analyses, a MID for the group with slight incontinence could not be determined. For the group with moderate severity the MID was determined to be 1.33 (95% CI 1.10–1.57) and for the severe/very severe group it was 3.58 (95% CI 3.08–4.09). Analysis of different types of incontinence showed no difference in MIDs.

**Conclusions:**

The MID for self-management via a mobile app was lower than previously established MIDs, but differed depending on baseline severity. This study shows that MIDs need adjustment for baseline severity and treatment intensity when interpreting clinical trial results. If using MIDs as exact numbers, the study population and the treatment must be comparable.

## Introduction

Urinary incontinence affects many women and has a large impact on the quality of life at a population level [1, 2]. Depending on how the leakage occurs, urinary incontinence symptoms are divided into three types: stress urinary incontinence (leakage upon exertion), urgency urinary incontinence (leakage associated with urgency) and mixed urinary incontinence (leakage both upon exertion and with urgency) [3]. First-line treatment includes pelvic floor muscle training (PFMT) and lifestyle advice for all three types [4, 5]. 

To measure improvement after treatment, the use of validated patient reported outcome measures (PROM) is recommended [6]. The most commonly used PROM for measuring symptoms is the International Consultation on Incontinence Questionnaire – Urinary Incontinence Short Form (ICIQ-UI SF) [7]. It is validated in terms of construct and convergent validity and reliability [8]. 

Responsiveness is another psychometric property that needs validation [6, 9]. One way of assessing the responsiveness is by establishing a Minimum Important Difference (MID) [6]. MID is the smallest change that can be considered meaningful to the patient and can be determined either through an anchor-based or through a distribution-based approach [6]. According to the COSMIN guidelines responsiveness is considered to express longitudinal validation [9]. The guidelines offer a checklist to assess quality when comparing one PROM with another outcome measurement [9]. 

The MID of ICIQ-UI SF has been evaluated in women with stress urinary incontinence after surgery [10], after pulsed magnetic stimulation [11] and after PFMT and lifestyle advice [12]. The MID varied from -4.5 to -5.7 points one year after surgery [10]. However lower MIDs were seen for conservative management; 4 points one year after pulsed magnetic stimulation [11] and 2.5 points after four months of PFMT and lifestyle advice [12]. These differences in MIDs may depend on the different populations and the intensity of the treatment. To our knowledge, the responsiveness of the ICIQ-UI SF has not been evaluated in any real-world studies. Real-world studies is a diverse field but refers to data collected outside of the traditional clinical trial context [13], like this study of self-management via a freely available mobile app without contact with health care professionals or study personnel. Furthermore, it has not been evaluated based on incontinence severity nor for all types of urinary incontinence.

The aim of this study was to analyse the responsiveness of the ICIQ-UI SF by determining a MID when women self-manage their urinary incontinence via a mobile app. The aim was also to analyse whether MID differs depending on the severity and type of incontinence.

## Methods

This study was a secondary analysis of data from an implementation study of the app Tät®, which was reported by Rygh et al. [14]. To study the real-world effect of the app Tät®, everyone who downloaded the app were informed about the study and asked to participate by completing a baseline and a follow-up questionnaire within the app. The baseline questionnaire included demographic questions, the purpose of downloading the app and the ICIQ-UI SF questionnaire. The app included information on stress urinary incontinence, lifestyle advice and training program for pelvic floor muscle training with visual support, reminders and statistical function. After 3 months the participants received a follow-up questionnaire as a pop-up in the app. It included questions on frequency of app use and PFMT, the questionnaires ICIQ-UI SF and Patient Global Impression of Improvement (PGI-I).

This current study included participants who downloaded the app from 16 January 2018 to 1 June 2019 with the intention of improving their urinary incontinence, and who submitted the follow-up questionnaire within 89–135 days. If the follow-up questionnaire was submitted after more than 135 days, the participant was not included in these analyses as it was deemed likely to reflect a more random use of the app than the continuous use that we aimed to study. Further inclusion criteria were female gender (self-defined), age 18–98 years and urinary incontinence at baseline. Urinary incontinence was defined as reporting any leakage on the ICIQ-UI SF question “How often do you leak urine?” and reporting any amount on the ICIQ-UI SF question “How much urine do you usually leak?”. All participants that submitted the follow-up questionnaire were included, regardless of whether they had performed PFMT with the app or not, but no questionnaire was sent if the user did not open the app. Table 1 describes to which extent the participants had reported to perform PFMT and use the app.

**Table 1:** Baseline and follow-up characteristics after 3 months of self-managing urinary incontinence via a mobile app (*n* = 1,733)

				All participants	Participants by incontinence severity		
					**Slight**	**Moderate**	**Severe/very severe**
Baseline factors							
	**Age, mean (SD)**			46.50 (13.29)	45.21 (13.36)	46.51 (13.22)	47.93 (13.34)
	**Education, n (%)**						
		≤ 9 years of school		47 (2.7)	6 (1.7)	26 (2.5)	15 (4.7)
		10–12 years of school		380 (21.9)	64 (18.1)	214 (20.2)	102 (31.9)
		University or college		1 306 (75.4)	284 (80.2)	819 (77.3)	203 (63.4)
	**Dwelling, n %**						
		Rural area		299 (17.3)	52 (14.7)	188 (17.8)	59 (18.4)
		Urban area < 50 000 inhabitants		461 (26.6)	92 (26.0)	276 (26.1)	93 (29.1)
		Urban area 50 000–1 million inhabitants		671 (38.7)	148 (41.8)	397 (37.5)	126 (39.4)
		Metropolitan area ≥ 1 million inhabitants		302 (17.4)	62 (17.5)	198 (18.7)	42 (13.1)
	Type of urinary incontinence, n %*						
		Stress Urinary Incontinence		949 (54.8)	229 (64.7)	583 (55.1)	137 (42.8)
		Mixed Urinary Incontinence		549 (31.7)	74 (20.9)	335 (31.6)	140 (43.8)
		Urgency Urinary Incontinence		188 (10.8)	45 (12.7)	108 (10.2	35 (10.9)
		Other Urinary Incontinence		47 (2.7)	6 (1.7)	33 (3.1)	8 (2.5)
	**ICIQ-UI SF, mean (SD)**			8.92 (3.77)	4.39 (0.69)	8.60 (1.97)	14.99 (1.92)
Factors at follow-up							
	**ICIQ-UI SF, mean (SD)**			7.50 (3.84)	4.51 (2.36)	7.20 (3.05)	11.81 (3.76)
	**Frequency of PFMT, n (%)**						
		No, never		154 (8.9)	41 (11.6)	79 (7.5)	34 (10.6)
		Less than once a week		401 (23.1)	96 (27.1)	236 (22.3)	69 (21.6)
		1–6 times a week		639 (36.9)	115 (32.5)	415 (39.2)	109 (34.1)
		Every day		433 (25.0)	84 (23.7)	268 (25.3)	81 (25.3)
		Three times a day or more		106 (6.1)	18 (5.1)	61 (5.8)	27 (8.4)
	**Usage of the app, n (%)**						
		Have not used it at all		185 (10.7)	44 (12.4)	98 (9.3)	43 (13.4)
		About once a month		231 (13.3)	50 (14.1)	141 (13.3)	40 (12.5)
		About once a week		368 (21.2)	76 (21.5)	235 (22.2)	57 (17.8)
		About once a day		489 (28.2)	105 (29.7)	308 (29.1)	76 (23.8)
		Several times a day		460 (26.5)	79 (22.3)	277 (26.2)	104 (32.5)

*Type of urinary incontinence based on the last item of the ICIQ-UI SF questionnaire

*SD* Standard Deviation, *PFMT* Pelvic Floor Muscle Training, *ICIQ-UI SF* International Consultation on Incontinence Questionnaire ? Short Form, *PGI-I* Patient Global Impression of Improvement

Exclusion criteria were pregnancy or recent delivery (partum within the last three months) at inclusion or follow-up.

The app Tät® is CE-marked as a medical device class 1, according to European Union regulation MDR 2017/745. To ensure accordance with the Declaration of Helsinki, the implementation study was approved by the Regional Ethical Review Board, Umeå (number 2012-325-31 M with amendments number 2014-389-32 M, 2016-80-32 M, 2017-405-32 M and 2020–04898) and the specific analysis conducted in this study was approved by the Swedish Ethical Review Authority (number 2020–04898). Informed consent was provided by all participants by ticking a box and submitting the questionnaires after reading the study information. To ensure data security all data was submitted anonymously.

### Outcome measures

This study used the ICIQ-UI SF, a validated PROM for symptom evaluation [8]. It includes three items asking about the frequency, amount and impact on everyday life of the urinary leakage. These questions form an additive score (range 0–21) which can be further categorized into severity categories (1–5 points = slight, 6–12 points = moderate, 13–18 points = severe, 19–21 points = very severe) [15]. 

The PGI-I is a validated, single-item PROM. It asks the person to rate their condition now compared with how it was before treatment [16]. In this study the participant was asked how their urinary leakage is now, compared with how it was before they downloaded the app Tät®, the seven possible answers ranged from “Very much better” to “Very much worse”. As only a few participants experienced deterioration, the categories “a little worse”, “much worse” and “very much worse” were collapsed into one category “worse”, for all answer categories see Table 2.

**Table 2:** Mean reduction in symptom score for each category of PGI-I at 3-month follow-up

PGI- I	N	ICIQ- UI SF
Very much better	133	3.78 (3.17–4.40)
Much better	304	2.43 (2.09–2.77)
A little better	722	1.46 (1.26–1.67)
No change	538	0.41 (0.16–0.65)
Worse	36	-1.58 (-2.72 - -0.44)

Values are means (95% confidence interval), *P* < 0.001 between groups. *N* = 1,733

### Sub-groups

For sub-group analysis the participants were divided into groups based on their ICIQ-UI SF score according to the severity categories described above [15]. To have an adequate number of participants for post hoc analysis, the groups with severe and very severe urinary incontinence were collapsed into one group.

A second division was then performed into sub-groups according to type of incontinence. The type was determined based on the last ICIQ-UI SF item according to the classification previously used by Espuña-Pons et al. [17] and as described in the article by Rygh et al. [14]. In accordance with current classification of symptoms [3], participants who reported urinary leakage when coughing or sneezing and/or upon exertion and who did not report leakage before they reached the toilet were categorised as having stress urinary incontinence. Women who indicated that they leaked before they reached the toilet but not when coughing or sneezing or when exercising were categorised as having urgency urinary incontinence. Those who reported urinary leakage in both cases were considered to have mixed urinary incontinence.

### Statistical analysis

The correlation between the PGI-I and the ICIQ-UI SF was analysed using the Spearman rank correlation. After establishing the correlation, we performed one-way ANOVAs to determine whether the mean scores at inclusion and the mean post-treatment reductions in scores on the ICIQ-UI SF were significantly different between different PGI-I categories. Welch’s ANOVA was used, when there was a significant difference in the homogeneity of variance between groups. If a significant difference was seen, Tukey’s post hoc test was used to analyse differences between group pairs. Differences were considered to be significant when *p* < 0.05. These analyses were performed for all participants and then in the sub-groups of participants with different severity and type of urinary incontinence.

MID was established using an anchor-based method. The mean reduction in ICIQ-UI SF of the group that responded to be “a little better” was considered to resemble MID, if this value was significantly different from the mean of the group that reported “no change”.

Due to the electronic submission of data, all participants had submitted complete baseline and follow-up questionnaires, hence there was no missing data. If the questionnaires were not fully completed, they could not be submitted and these participants could not be included in this study. SPSS version 26 was used for all. analyses.

## Results

This study included 1,733 women with all types of urinary incontinence. Of these, 54.8% answered that they only had symptoms of stress urinary incontinence, 10.8% only reported symptoms of urgency urinary incontinence, and 31.7% reported symptoms of both types and were categorized as having mixed urinary incontinence. The participants’ mean age was 46.5 years, ranging from 19 to 87 (interquartile range 36–55), and 75.4% had a higher level of education (university or college).

At baseline the mean ICIQ-UI SF score was 8.92 (range 3–21). According to the severity categories established by Klovning et al. [15], 20.4% (*n* = 354) had slight severity, 61.1% (*n* = 1,059) had moderate, 17.3% (*n* = 300) had severe and 1.2% (*n* = 20) had very severe. At follow-up the mean ICIQ-UI SF score was 7.50 (range 0–21). The means of the different severity groups, additional baseline and compliance data are available in Table 1.

**Fig. 1:**
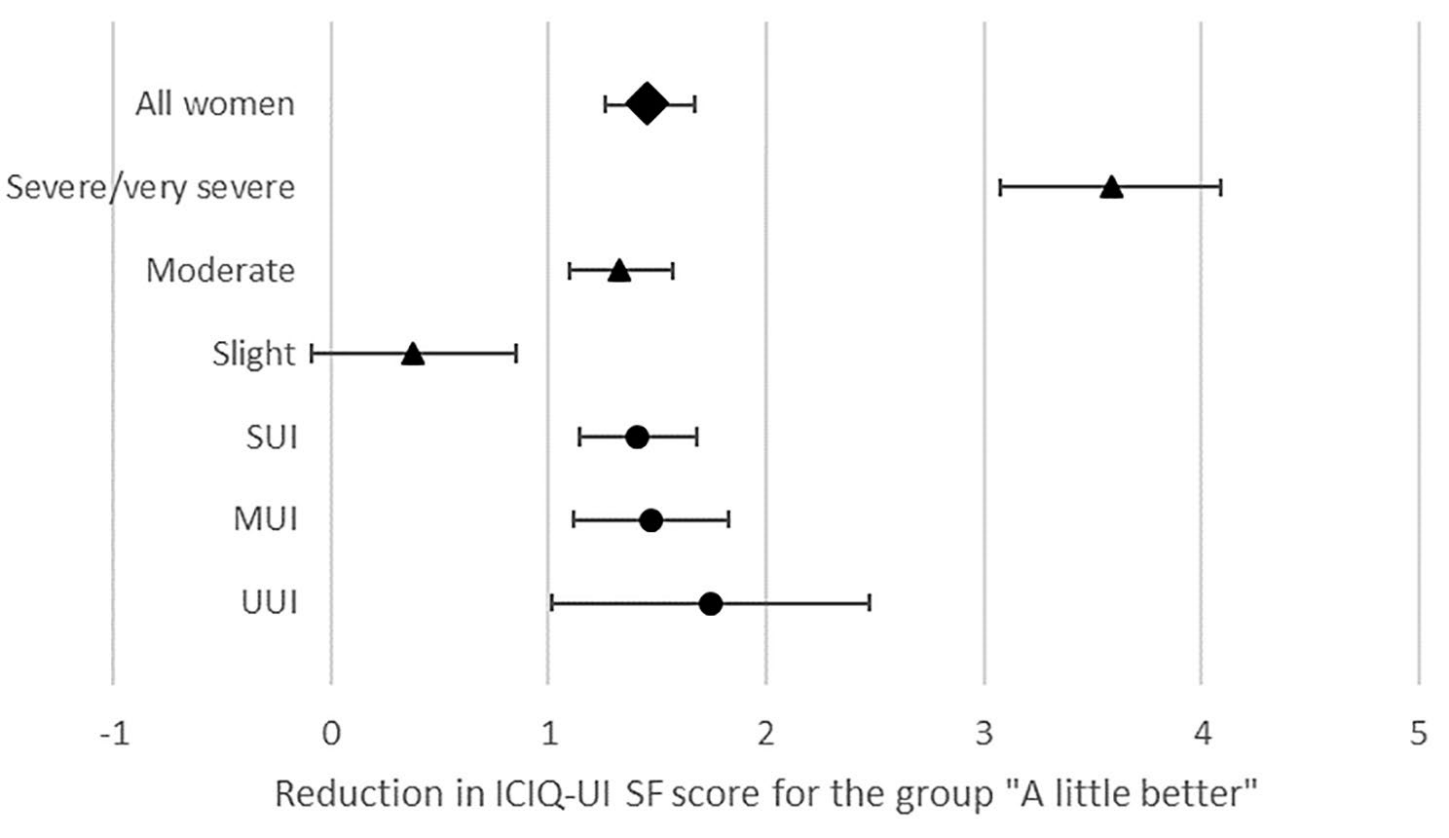
Mean reduction in ICIQ-UI SF score for the group who stated being “a little better” among all women and in the analysed sub-groups. Subgroups were analysed according to incontinence severity, marked with a triangle, and according to incontinence type, marked with a circle. The error bar indicates 95% confidence interval. For exact means and confidence intervals, please see Tables 2, 3 and 4. SUI: Stress Urinary Incontinence, MUI: Mixed Urinary Incontinence, UUI: Urgency Urinary Incontinence

For the total group there was a significant correlation between the PGI-I and change in ICIQ-UI SF, Spearman rho − 0.323 (*p* < 0.001). The correlation between PGI-I and post-treatment ICIQ-UI SF score was 0.306 (*p* < 0.001). The one-way ANOVA revealed significant differences between all groups with *p* ≤ 0.001. (Table 2) The mean reduction for the group who stated being “a little better” was 1.46 (95% Confidence Interval (CI) 1.26–1.67) which was set to be the minimum important difference (MID). Figure 1 shows the mean change in ICIQ-UI SF score for the group “a little better” in all women and in the different sub-group analysis.

**Table 3:** Mean reduction in symptom score for each category of PGI-I and severity category at 3-month follow-up

		Slight				Moderate				Severe/very severe			
PGI- I		N		ICIQ- UI SF		N		ICIQ- UI SF		N		ICIQ- UI SF	
Very much better		35		1.74 (0.80–2.68)		81		4.07 (3.35–4.80)		17		6.59 (4.40–8.78)^c^	
Much better		69		0.38 (-0.09–0.85)^a^		184		2.61 (2.22–3.00)		51		4.55 (3.53–5.57)^c, d^	
**A little better**		**125**		**-0.25 (-0.57–0.07)** ^**a**^		**467**		**1.33 (1.10–1.57)**		**130**		**3.58 (3.08–4.09)** ^**d, e**^	
No change		114		-0.49 (-0.88 - -0.10)^a^		308		0.24 (-0.08–0.56)^b^		116		1.73 (1.15–2.32)^f^	
Worse		11		-3.82 (-5.92 - -1.72)		19		-1.26 (-2.46 - -0.06)^b^		6		1.50 (-2.52–5.52)^e,f^	
Total		354				1059				320			

Values are means (95% confidence interval), *P* < 0.05 between groups. *N* = 1,733

^a−f^ No significant differences between these categories. For example, among participants with slight incontinence there was no difference between the groups expressing “no change”, “a little better” and “much better”, so these are all marked with ^a^

**Table 4:** Mean reduction in symptom score for each category of PGI-I and type of urinary incontinence at 3-month follow-up

		Stress Urinary Incontinence				Mixed Urinary Incontinence				Urgency Urinary Incontinence			
PGI- I		N		ICIQ- UI SF		N		ICIQ- UI SF		N		ICIQ- UI SF	
Very much better		76		3.87 (3.08–4.66)		38		3.03 (1.72–4.33)^a^		15		4.60 (3.05–6.15)	
Much better		150		2.48 (1.96–3.00)		103		2.35 (1.78–2.91)^a, b^		40		2.25 (1.49–3.01)^e^	
**A little better**		**409**		**1.41 (1.14–1.68)**		**222**		1.47 (1.11–1.83)* ^**b,c**^		**73**		**1.74 (1.01–2.47)** ^**e**^	
No change		298		0.43 (0.12–0.73)		170		0.39 (-0.11–0.89)^d^		57		0.02 (-0.57–0.61)^f^	
Worse		16		-2.19 (-3.77 - -0.60)		16		-0.38 (-2.02–1.27)^c, d^		3		-3.00 (-16.14–10.14)^f^	
Total		949				549				188			

Values are means (95% confidence interval), *P* < 0.05 between groups. *N* = 1,733

^a−f^ No significant differences between these categories

*Due to the low correlation (< 0.3) with PGI-I, this should not be considered as a MID.

For the group with slight incontinence at baseline, there was also a significant correlation, Spearman rho − 0.318 (*p* < 0.001), between the PGI-I and change in the ICIQ-UI SF score. The one-way ANOVA showed that there was significant differences within the ANOVA (Welch test *p* < 0.001) but in the post hoc analysis there were no differences at the *p* < 0.05 level between the groups “no change”, “a little better” and “much better”. (Table 3) Hence, a MID could not be established. These three groups were however significantly different from the groups of participants who expressed being “very much better” and “worse”.

For the group with moderate incontinence the correlation between the PGI-I and change in the symptom score was slightly stronger, Spearman rho − 0.369 (*p* < 0.001). The ANOVA revealed significant differences between all groups who reported no change or improvement. The mean value of the group who answered that they were “a little better” was 1.33 (95% CI 1.10–1.57). (Table 3)

In the group with severe or very severe urinary incontinence the correlation between the PGI-I and the difference in the ICIQ-UI SF was − 0.379 (*p* < 0.001). The ANOVA showed significant differences between the groups, Welch test *p* < 0.001. Groups were smaller and the post hoc tests showed that there were no significant differences between the different categories and the closest answer categories, except between “no change” and “a little better” (*p* < 0.001). This meant that the mean of the “a little better” group, 3.58 (95% CI 3.08–4.09), was significantly different from “no change” and could be used as a MID.

In the analysis of different types of incontinence there were significant correlations of similar strength for stress (rho=-0.350, *p* < 0.001), mixed (rho=-0.274, *p* < 0.001) urgency (rho=-0.435, *p* < 0.001) and other urinary incontinence (rho=-0.398, *p* = 0.006). The ANOVAs for stress, mixed and urgency incontinence were significant with *p* < 0.001. Post hoc tests according to Tukey showed significant differences for the groups “a little better” and “no change” within each group. The mean for the group “a little better” was not different between types. (Table 4) In the group with other incontinence, the ANOVA showed that there was a significant difference between the groups (*p* = 0.01) but the groups were too small to perform post hoc analysis and it was not considered meaningful to collapse them further.

## Discussion

### Main findings

This study confirmed that women with larger symptom score reductions experienced greater improvements. At a group level, a reduction of 1.46 points on the ICIQ-UI SF score would be the minimum important difference after self-management of urinary incontinence via eHealth. For women with slight incontinence, the variation was large and the MID could not be determined although the sample size was large. This suggests that other measures, for example the PGI-I, should be used to determine clinical relevance in this group. For the other severity groups, the MID was set to 1.33 points for women with moderate incontinence, and to 3.58 points for women with severe/very severe incontinence. Regarding different types of urinary incontinence, no differences in the MID were found.

### Strength and limitations

The strengths of this study include the large sample size and the fact that the participants filled out the questionnaire without any influence from the researchers. Additionally, the electronic questionnaires did not allow for data to be omitted. Further strengths included the use of validated and commonly used patient reported outcome measures, and using the PGI-I within the validated time frame to minimize the risk of incorrect recollection. Also, previous studies have shown effect of PFMT-focused intervention within the timeframe. These are all factors listed by the COSMIN guidelines to ensure adequate quality [9]. 

The most important limitation is the real-world setting of complete self-management, which means that the MID for the general group cannot be generalized to other study settings that include more intense evaluation, instructions and follow-up. These MIDs from a real world setting rather provides further knowledge on how MIDs could need adjustment according to severity at start.

Furthermore, the study group was well-educated which may have affected the impression of improvement. Other studies have shown that women with a higher socioeconomic status are less likely to be satisfied with treatment [18] and women with a higher level of education are more likely to cross over to surgery [19]. Another limitation was that the type of urinary incontinence was based on the self-report in the last ICIQ-UI SF item and hence the diagnosis of type might not be correct in all cases.

### Comparison with previous studies

The correlation found in our study (*r*=-0.323) is slightly weaker than in previous studies of conservative management (*r* = 0.547) [11] and studies of surgery (*r* = 0.43 for stress urinary incontinence and *r* = 0.48 for mixed urinary incontinence) [20]. These studies correlated change in score and not reduction, hence the positive correlation. This could support the recommendation that different aspects including quality of life and patient goals need to be taken into account for a comprehensive evaluation [21]. A qualitative study of another app for urinary incontinence management also revealed that using the app could also increase awareness of symptoms [22], which could also interfere with the correlation as symptoms are self-reported. The variation of compliance, both app use and PFMT frequency, could also contribute to the weaker association.

Our results present a lower MID for conservative management compared with previous studies [11, 12]. This could be partly explained by the differences in baseline severity found in this present study, whereas previous studies included women with a higher severity. It may also be explained by different expectations, the intensity of treatment, the invested effort and the lower treatment effect seen in our study. In our study, almost a third of the participants had not performed PFMT regularly during the last month prior to follow-up, and there were no clinical visits or other efforts required of the participants. In contrast, the other studies included more contact with researchers [12] and more clinical visits [11] or even surgery [10]. The study by Lim et al. also displays a range of MIDs depending on which anchor is used, whereas we used a different anchor-based method [11]. 

The group with slight urinary incontinence at baseline is a group rarely included in studies. After surgery participants with such mild symptoms have even been considered to be cured [20]. Therefore it is difficult to say which improvement that could be anticipated in this group. A previous study of this population has shown larger treatment effects for higher baseline severity [14], but no previous studies have described that the ICIQ-UI SF score would be less responsive at either end of the scale, and that the MID would need to be adjusted accordingly. If the initial score is low (in the group “slight” the maximum score is 5 points), large reductions in score are not possible and the score may be more influenced by the perceived bother if the leakage is small. In this study, there was no change in mean score from baseline to follow-up. This could at least partly explain why no exact relationship could be determined with the impression of improvement.

To our knowledge, no studies have evaluated the MID for the ICIQ-UI SF in women with mixed and urgency urinary incontinence. Our results indicate that there is no difference in the MID between different types of urinary incontinence. The ICIQ-UI SF only asks for symptoms of incontinence and not for symptoms of overactive bladder. However, the bother scale accounts for almost half of the score (10 of 21 possible points) meaning that symptoms of frequency and urgency may still be included in the total assessment of bother.

All methods for establishing a MID have their limitations. We have chosen a patient-centred approach by using a global rating scale as an anchor, which is sometimes recommended [23]. We find the anchoring to the group that could detect a small difference, as described by Jaeschke et al. [24], to be an intuitive and most reasonable approach. While others have arrived at different conclusions [10, 11], their results and the results of this study show that there is no exact value.

### Implications of findings

This study established MIDs that can be used at group level, for low-intensity treatments such as self-management via a mobile app. For treatments with higher intensity, other previously established MIDs should be used. However, our results also show that if baseline severity is higher, larger reductions are needed to perceive improvement. This is most likely generalizable to other settings meaning that previously established MIDs may also need adjustment for baseline severity when applied to new groups. This study offers no support that adjustment of MID is needed based on type of urinary incontinence.

The results also show that there are large individual variations between women and thus underline the need to both evaluate the change in symptoms and perform a comprehensive evaluation. Therefore, the use of MID in the group with slight severity or on an individual level cannot be considered useful.

## Conclusion

After real-world self-management of urinary incontinence with a mobile app, minimum important differences are lower than with other conservative management. If baseline incontinence is more severe, a larger reduction is needed for clinical relevance.

## Data Availability

The dataset analysed during the current study is available from the corresponding author on reasonable request.

## Abbreviations

ANOVA: Analysis of Variance
CI: Confidence Intervals
ICIQ–: UI SF International Consultation on Incontinence Questionnaire–Short form
MID: Minimum Important Differences
PGI–: I Patient Global Impression of Improvement
PFMT: Pelvic Floor Muscle Training
PROM: Patient Reported Outcome Measure
